# Bronchial obstruction secondary to idiopathic scoliosis in a child: a case report

**DOI:** 10.1186/1752-1947-2-171

**Published:** 2008-05-22

**Authors:** Saad Alotaibi, James Harder, Sheldon Spier

**Affiliations:** 1 Alberta Children's Hospital, University of Calgary, AB, Canada

## Abstract

**Introduction:**

Patients with severe idiopathic scoliosis are reported to have significant pulmonary complications, including recurrent chest infections, alveolar hypoventilation and respiratory failure.

**Case presentation:**

We report a case of a 13-year-old boy with moderate-to-severe scoliosis resulting in torsion or twisting of the bronchus intermedius, which contributed to airflow obstruction defects, as revealed by both spirometry and bronchoscopy.

**Conclusion:**

We recommend that inspection of the shape of the maximal expiratory flow-volume loop obtained from spirometry, as well as other parameters suggestive of obstructive lung disease, may be important in children with scoliosis. To the best of the authors' knowledge, this is the first report of a child in which pulmonary function testing and direct visualization via a flexible bronchoscope have been used to characterize intrathoracic large airway obstruction.

## Introduction

Scoliosis can be acquired or idiopathic. Acquired scoliosis has no definite curve pattern. Idiopathic scoliosis is the commonest type of scoliosis and is usually found in young people including children. Radiography is the most objective method of examining the scoliotic spine. Curve assessment is done frequently by Cobb's curve measurement on the radiographs to determine the extent of its progression. Most previous studies have shown that patients with idiopathic scoliosis have a restrictive lung defect. Obstructive lung disease on the other hand was believed not to be associated with idiopathic scoliosis. In this case report we document obstruction of the airways using both spirometry (PFT) and flexible bronchoscopy.

## Case presentation

**Figure 1:**
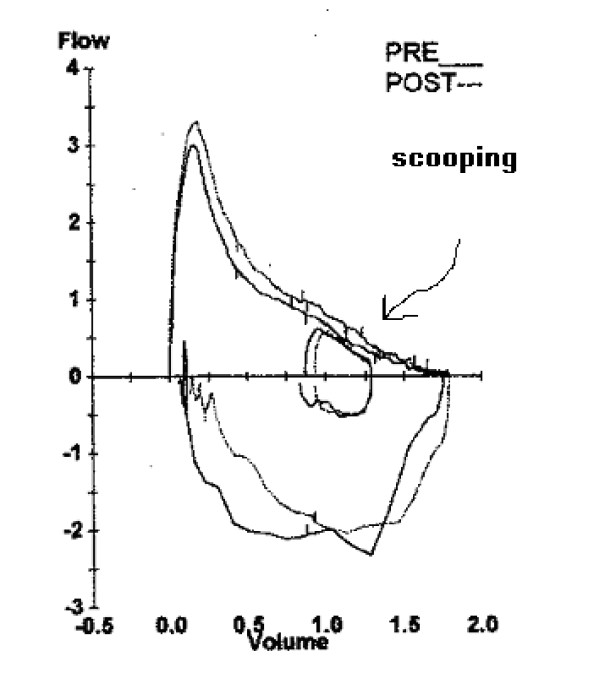
**Pulmonary function test.** The pulmonary function test was performed pre-operatively; no spirometric improvement was noted after the administration of pre- and post-bronchodilator therapy. There was scooping and concavity of the expiratory part of the flow-volume curve (arrow), which indicates obstructive airway disease. Spirometry revealed a moderate-to-severe reduction in forced vital capacity, forced expiratory volume at 1 minute, forced expiratory volume 25% to 75%, and total lung capacity. There was evidence of hyperinflation and airway trapping from the increase in functional residual capacity, residual volume and airway resistance.

A 13-year-old boy presented with severe scoliosis, characterized by a convexity to the right and significant rotation. He had a previous placement of sub-cutaneous titanium rods extending from T3-4 to L1-2 at 10 years of age after failure of bracing. His pulmonary function test (PFT) revealed a scooped flow-volume curve with a fixed moderate-to-severe obstructive respiratory defect and evidence of air trapping (Figure 1, Table 1). The PFT was performed prior to bronchoscopy at 13 years of age as a routine investigation in all patients at the authors' institution. Overnight pulse oximetry was normal. His pre-operative blood gas was normal with no CO_2 _retention. He had no history of asthma.

**Table 1:** Primary Function test

Parameter	**Ref**.	**Pre. Meas**.	**Pre. % Ref**.	**Post. Meas**.	**Post. % Rf**.	Post % Change
FVC (Liters)	2.59	1.76	68	1.79	69	2
FEV1 (L)	2.36	1.13	48	1.23	52	9
FEV1/FVC%	86	64		69		
FEF25-75% L/sec.	2.75	0.67	24	0.82	30	22
TLC (L)	3.34	2.98	89	2.95	88	-1
FRC PL (L)	1.56	1.93	124	1.93	124	0
RV (L)	0.7	1.19	169	1.12	159	-6
RV/TLC %	21	40		38		
Raw cmH2O/L/sec	3.49	7.65	220	4.54	130	-41
sRaw cmH2O/L/s/L	5.43	15.67	288	8.75	161	-44

Previous medical history revealed a birth weight of 2.4 kg and an uneventful infancy. Brace treatment with a thoraco-lumbar-sacral-orthosis was started at 5 years of age, but failed to prevent progression of the curve. He had a laminectomy and release of a tethered spinal cord when he was 10 years of age. Spinal rods without fusion were placed when he was 11 years of age. At that time, his degree of scoliosis, as measured by the Cobb's angle, was 68°, with a 15° rotation. He had a chronic cough, respiratory distress with upper respiratory viral illnesses, and frequent episodes of atelectasis and recurrent pneumonias involving the right middle and lower lobes. His serial chest radiographs demonstrated collapse of the right lower lobe, hyperinflation and a scoliosis deformity. He was on salbutamol nebulizer as needed, budesonide nebulization when he developed viral illnesses, and antibiotics with episodes of pneumonia. There was no evidence of pulmonary hypertension based on clinical examination, so an echocardiography was not performed.

**Figure 2:**
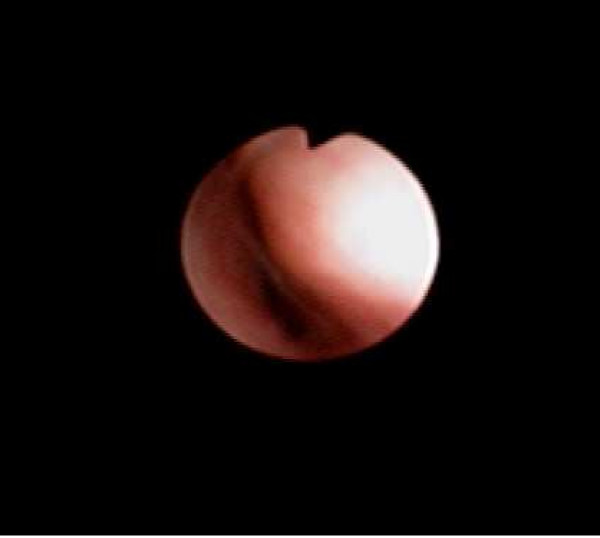
Flexible bronchoscopy demonstrated a compression of the right lower and middle lobe bronchi with a slit-like appearance.

Physical examination revealed scoliosis and decreased air entry to the right hemithorax. Flexible bronchoscopy before surgery demonstrated a compression of the right lower and middle lobe bronchi with a slit-like appearance (Figure 2). This was due to torsion of the bronchus intermedius just distal to the right upper lobe bronchus. He had a distraction of his spinal rod instrumentation to partially correct the scoliosis, which improved his Cobb's angle to 38°. The postoperative course in the intensive care unit was uneventful and he was transferred to the ward 2 days later. He spent 10 days on the ward receiving analgesia and chest physiotherapy. No stents were placed and the orthopedic surgeon believed that de-torsion would occur after correction of the scoliosis.

## Discussion

Deformities of the dorsolumbar spine are the most common cause of symptomatic deformities of the chest wall. Scoliosis consists of lateral angulation and rotation of the spine and is categorized as right (most frequently) or left, according to the direction of the convexity of the curvature [1]. The severity of scoliosis is quantified by measuring the angle (that is, Cobb's angle) between the upper and lower portions of the spinal curve on a radiograph. Any abnormality of respiratory function is detectable only when this angle exceeds 70° (see [1]). However, one study has found restrictive and/or obstructive airway abnormalities in patients with scoliosis of less than 60°, even though many of these patients were asymptomatic [2]. Adolescent idiopathic scoliosis consists of a lateral and rotational spinal curvature in the absence of associated congenital or neurologic abnormalities. Longitudinal studies [3,4] have estimated the prevalence of idiopathic scoliosis as 2% of the adolescent population, using a definition of a spinal curve as greater than 10°. However, clinically significant curves in the range of 40° to 100° are rare [5]. The incidence of brainstem or spinal cord anomalies, such as tethered cord, in patients with idiopathic scoliosis ranges from 4% to 58% (see [6]).

Several therapeutic approaches for scoliosis are available. First-line treatment is thoracolumbar orthosis, which is placed in an effort to prevent further increase in the curve with growth. If there is progression of the curve with spinal orthosis, the usual recommendation includes insertion of spinal rods without fusion in the growing child. Further surgical correction may include rods with spinal fusion [1]. Sakiæ et al. [7] reported that scoliosis only affects pulmonary function in the upper thoracic curves when the apex between T5 and T8 exceeds 70°, and in such cases there is a direct correlation between vital capacity (VC) and increased curve severity. They observed a significant improvement of cardiopulmonary function after spinal stabilization and correction was observed after 2 years. Further, they noted that a 54% surgical correction was correlated with an increase of VC, forced expiratory volume at 1 minute (FEV1), maximum midflow at 25% to 75% VC, functional residual capacity, total lung capacity, and improved exercise tolerance [7].

The patient had scoliosis with a Cobb's angle of 68° and 15° rotation. Clearly there was intrathoracic airway obstruction based on the spirometry, which was confirmed by bronchoscopy. However, previous studies have shown that patients with idiopathic scoliosis have a restrictive lung defect [1]. Obstructive lung disease, on the other hand, was believed not to be associated with idiopathic scoliosis. Weber et al. [8] found no evidence of airway obstruction based on the FEV1/VC, closing volume, and expiratory flow rate at 50% of VC. However, Boyer et al. [2], in their review of pulmonary function of 44 children with idiopathic scoliosis before surgical correction, found that 46% had moderate-to-severe gas trapping and 23% had mild gas trapping. They hypothesized that this was indicative of obstructive airway disease. However, this may be caused by mechanical restriction of the thoracic cage to forced expiration. Nevertheless, the improvement in specific conductance they noted after administration of a bronchodilator may indicate airway obstruction. The authors did not describe the shape of the flow-volume curves in their patients, which may reveal any scooping or concavity of the expiratory part of the curve.

Airway obstruction and gas trapping may increase the peri-operative risk of atelectasis and pneumonia with subsequent ventilation-perfusion mismatching and impaired alveolar gas exchange. Analysis of the shape of the flow-volume loop can distinguish variable from fixed obstruction as either superior or inferior to the sternal notch [9,10]. This obstruction could be due to either compression by the vertebral bodies or true twisting or torsion [11]. Al-Kattan et al. [11] reported three adults with severe kyphoscoliosis leading to bronchial torsion and obstruction of the central airways. Patients with scoliosis treated surgically by instrumentation and fusion in an attempt to correct the spinal deformity have shown improvement in both functional VC and FEV1 [7,12]. These findings suggest that severe kyphoscoliosis with a chest wall deformity could affect the VC as well as cause central airway obstruction causing a reduction in the forced expiratory volume in some patients. The severity or the angle of the scoliosis was neither a good predictor of the site, nor the side of the torsion. In a case report involving a teenage girl with scoliosis, Borowitz et al. [13] described flattening of the initial portion of the expiratory loop, suggesting fixed obstruction of the large airways, which showed marked improvement in the shape of the flow-volume loop after surgical correction of the scoliosis. However, they did not comment on the lung volume-dependent portion of the curve, which indicates intrathoracic events and was unchanged after intervention. This may suggest multiple levels of obstruction.

## Conclusion

We suggest evaluation of children with scoliosis using spirometry and a flow-volume curve to determine whether there is evidence of airway obstruction. If there is obstruction, direct visualization through a flexible bronchoscope would help to identify the precise site and severity of the airway obstruction and guide further management such as spinal orthosis or insertion of spinal rods. As reported here, there is clinical significance to this observation and more data and research are needed to reach definitive conclusions about restrictive obstructive airway disease in childhood idiopathic scoliosis.

## Abbreviations

FEV1: forced expiratory volume at 1 minute; PFT: pulmonary function test; VC: vital capacity.

## Competing interests

The authors declare that they have no competing interests.

## Authors' contributions

SA collected the data about the patient, performed the literature search and prepared the draft for publication. SS interpreted the spirometry and bronchoscopy results and helped in revising the draft with important clinical input. JH helped with revising the data about idiopathic scoliosis and provided data about orthopedic management of such cases and helped in critically revising the manuscript for publication.

## Consent

Written informed consent was obtained from the patient for publication of this case report and any accompanying images. A copy of the written consent is available for review by the Editor-in-Chief of this journal.
